# Study of Water Resistance of Polyurethane Coatings Based on Microanalytical Methods

**DOI:** 10.3390/polym16243529

**Published:** 2024-12-18

**Authors:** Chao Xie, Yufeng Shi, Zhuozhuo Si, Ping Wu, Binqiang Sun, Wenzhe Ma

**Affiliations:** 1 Civil Engineering Department, Lanzhou Jiaotong University, Lanzhou 730070, China; shiyf25260@163.com (Y.S.); 13639526849@163.com (Z.S.); m1124278455@163.com (W.M.); 2 CSCEC AECOM Consultants Co., Ltd., Lanzhou 730000, China; y252666@163.com; 3 Gansu Yi’an Construction Technology Group Co., Ltd., Lanzhou 730060, China; sunbq8882023@163.com

**Keywords:** polyurethane coatings, microstructure, diluent content, water resistance, free volume, characteristic molecular groups

## Abstract

This study investigates the effect of microstructural changes in polyurethane coatings on their water resistance properties. Polyurethane coatings with varying diluent contents were prepared and tested for water penetration resistance and mechanical property retention. The time-dependent behavior of water within the coatings at different immersion durations was analyzed using low-field nuclear magnetic resonance (NMR). Furthermore, the free volume and characteristic molecular groups of each coating were analyzed using microscopic techniques, including positron annihilation lifetime spectroscopy (PALS) and attenuated total reflectance Fourier-transform infrared spectroscopy (ATR−FTIR). Results indicate that diluent content significantly alters the microstructure of the coatings. With increasing diluent content, both the average pore volume and free volume fraction initially decrease and then increase, while characteristic molecular groups, including hydrophilic groups, gradually decline. The water resistance performance of the coatings was significantly influenced by the combined effects of free volume and characteristic molecular groups. Among the five tested coating formulations, coatings with diluent contents of 20% and 25% showed a superior water penetration resistance, higher retention of mechanical properties after immersion, and relatively low total content of bound and free water at all immersion ages. The entropy weight method and the equal weight method were used to assess the overall water resistance, with the following ranking of scores: *f*_20_ > *f*_25_ > *f*_30_ > *f*_15_ > *f*_10_. This study offers theoretical support to guide the design and practical application of polyurethane coatings in real-world engineering projects.

## 1. Introduction

Concrete, as a fundamental material for infrastructure construction, is widely utilized in bridges, roads, tunnels, high-rise buildings, and water conservancy projects [1]. However, the durability of concrete structures has remained a focal point of research in the field of civil engineering. In practical engineering environments, corrosive salts penetrate the structure through moisture absorption, leading to the accelerated deterioration of concrete, which significantly compromises both the service life and safety of the structure [2,3]. To enhance the durability of concrete structures, protective coatings are commonly applied to their surfaces to shield them from external corrosive agents, thereby prolonging the service life of concrete. Among various organic coatings, polyurethane coatings are extensively used in the protection of concrete structures due to their superior mechanical properties and strong adhesion [4,5]. However, as polymers, the chemical structure and mechanical properties of polyurethane coatings are often significantly altered in high-humidity or underwater environments, thereby shortening their service life and diminishing their protective effectiveness [6,7]. Therefore, studying the water resistance of polyurethane coatings is crucial for predicting their protective effectiveness and service life in practical engineering applications.

Previous studies have demonstrated that the microstructure of polyurethane materials significantly influences their water resistance [8,9,10,11,12]. When the coating is exposed to a high-humidity environment, water molecules penetrate the polyurethane structure and form hydrogen bonds with polar groups, reducing the original intermolecular forces. This plasticizing effect increases the mobility of the polymer chains and lowers the glass transition temperature, consequently reducing hardness, strength, and abrasion resistance [12,13]. Additionally, the ester-based molecular segments in polyurethanes undergo hydrolysis reactions, increasing the free volume between the molecular chains. This results in a decrease in both the strength and toughness of the coating, thereby increasing the risk of coating cracking [11,14]. Therefore, the water resistance of polyurethane coatings in high-humidity environments is closely linked to changes in their microstructure, which, in turn, significantly affects their protective performance during service.

Although significant progress has been made in the study of water resistance in polyurethane coatings, most studies have primarily focused on the effect of changes in the chemical structure of the coating, such as molecular chain segments, on its water resistance. However, in real-world construction environments fluctuations in temperature and humidity often necessitate adjusting the viscosity of the coating by adding diluents to meet construction pace and process requirements. This process not only affects the characteristic functional groups of the coating but also alters its free volume and other microstructural properties, which in turn impact the water resistance of the coating [15,16]. However, there are few studies investigating the combined effect of these two microstructural properties on the water resistance of polyurethane coatings.

To address this research gap, this study designs polyurethane coating systems with varying viscosities by altering the diluent content and adjusting the microstructure of the coatings [15,16]. This study combines macroscopic water resistance indices (e.g., resistance to water infiltration and retention of mechanical properties after immersion) with nuclear magnetic resonance (NMR) analysis to systematically explore the water resistance development patterns of different coatings. The properties of different coatings were systematically investigated. Concurrently, the free volume and characteristic molecular groups of the coatings were analyzed using microscopic techniques (e.g., PALS, ATR−FTIR). The evolution mechanism of the water resistance of the coatings was explored to elucidate the correlation between water resistance and its microscopic performance indices. This correlation between water resistance and its microscopic performance indices is elucidated, providing both a theoretical basis and technical support for further optimization in the design and application of polyurethane coatings in practical engineering.

## 2. Test Materials and Methods

### 2.1. Test Raw Materials

The coatings employed in this study are BS52-40 polyurethane anticorrosive coatings and their diluent, provided by Xinjiang Youbaote Anticorrosive Coatings Co., Ltd. (Urumqi, China).

The test cement was 42.5-grade ordinary Portland cement (produced by Lanzhou Glycyrrhizin Cement Group, Lanzhou, China). The fine aggregate was ISO-standard sand (produced by Xiamen ASio Company, Xiamen, China), with a particle size range of 0.5–1.0 mm. The coarse aggregate consisted of 5–31.5 mm continuous-graded crushed stone, with a crushing index of 7.04% and an apparent density of 2792 kg/m^3^.

### 2.2. Preparation of Test Samples

(1) Coating specimens

On actual construction sites, diluents are commonly added to adjust the viscosity of coatings, in order to adapt to environmental conditions and construction process requirements. In this study, diluents were incorporated at concentrations of 10%, 15%, 20%, 25%, and 30% by weight of the coating to prepare the specimens. The coatings underwent the following curing process: first, they were cured at room temperature (20 °C ± 2 °C, 55% ± 3% humidity) for 12 h; next, they were placed in a curing chamber (23 °C ± 2 °C, 50% ± 3% humidity) for 7 days; and finally, they were returned to room temperature for an additional 7 days of curing. After several tests, the thickness of the cured coatings consistently measured approximately 1 mm. The diluent concentrations of 10%, 15%, 20%, 25%, and 30% correspond to coating sample numbers *f*_10_, *f*_15_, *f*_20_, *f*_25_, and *f*_30_, respectively.

The electric flux specimens were prepared based on the mix ratios provided in Table 1 and the specifications outlined in [17]. The relatively flat side of each specimen was selected and polished. After removing surface contaminants, the specimens were oven-dried at 50 °C ± 2 °C for 24 h. The prepared paint was then applied and allowed to cure according to the specified method. The electric flux test specimens, corresponding to the coating samples, are labeled D*_f_*_10_, D*_f_*_15_, D*_f_*_20_, D*_f_*_25_, and D*_f_*_30_, with the uncoated control sample labeled as D*_f_*_0_.

### 2.3. Test Design and Test Methods

(1) Film immersion test

The prepared coating samples were cut into rectangles measuring 20 mm × 100 mm, dried in an oven at (50 ± 2) °C for 48 h, and then sealed with wax around the coated specimens. The initial mass of the samples was then recorded. The samples were subsequently submerged in deionized distilled water, and the coating films reached a saturated state of water absorption after 14 days of immersion. The samples were then removed, and the surface water was dried. The mass of each sample was subsequently determined using a micro-electronic balance with an accuracy of 0.1 mg.

(2) Mechanical properties of the coating

The tensile strength and other mechanical properties of the coating were measured using a universal material testing machine (WDW-50, manufactured by EST Test Instruments), with the upper chuck moving at a speed of 50 mm/min. The coating sample prepared in Section 2.2 was cut into dumbbell-shaped specimens and divided into two groups for tensile strength testing. The first group was directly tested, and its post-fracture elongation was calculated based on the length change in the specimens before and after the test. The second group was soaked using the method described in Section 2.3–(1) and then tested for its tensile strength and elongation after fracture.

(3) Water penetration resistance of the coating

The “electric flux” method, as outlined in specification [17], was employed to assess the impermeability of each group of coated concrete composite specimens. The test results were then compared with those of uncoated specimens to further evaluate the water permeability resistance of each group of coatings.

(4) The microscopic distribution of water in the coating

As the soaking time increases, the internal structure of the coating and the state of water within it change. To further investigate the water resistance of the coatings, low-field nuclear magnetic resonance (NMR) was employed to analyze the various immersion stages. NMR technology allows for continuous monitoring of water distribution changes in the coating under non-destructive conditions, offering advantages such as non-destructiveness, continuity, and speed. The instrument used in this study is the MacroMR12-150H-I nuclear magnetic resonance system, manufactured by Suzhou Niumai Analytical Instrument Co., Ltd. (Suzhou, China) The magnetic field intensity during testing is 0.2 ± 0.05 T, with an operating frequency of 12.638 MHz. The test results were analyzed using Ver4.0 software.

(5) Characteristic molecular groups of the coating

The characteristic functional groups of the coating samples were analyzed using attenuated total reflection infrared spectroscopy (ATR−FTIR). The ATR infrared spectra of each group of coating samples were recorded using a Thermo Nicolet is5 infrared spectrometer (Waltham, MA, USA). The spectra were recorded over a range of 400–4000 cm^−1^, with a resolution of 8 cm^−1^. Each spectrum was obtained by co-adding 128 scans and analyzed using the OMNIC 8.2 software, which is provided with the instrument, to identify the characteristic molecular groups of each group of coating samples. The types and contents of the characteristic molecular groups in each group of coating samples were determined.

(6) Microstructure of the coating

Positron annihilation lifetime spectroscopy (PALS) is widely used to obtain microscopic information about polymers, and, therefore, in this study PALS is employed to analyze the microstructure of different coating samples [18]. The positron source used is ^22^Na, with an intensity of approximately 13 μCi. The gamma rays emitted during positron annihilation are detected by a pair of BaF2 scintillator detectors. A total of 2 million positron annihilation lifetime spectra were collected, with a time resolution of approximately 195 ps, and the spectral data were analyzed using LT9.0 software.

## 3. Results and Discussion

### 3.1. Water Resistance of the Coating

#### 3.1.1. Water Penetration Resistance of the Coating

**Figure 1:**
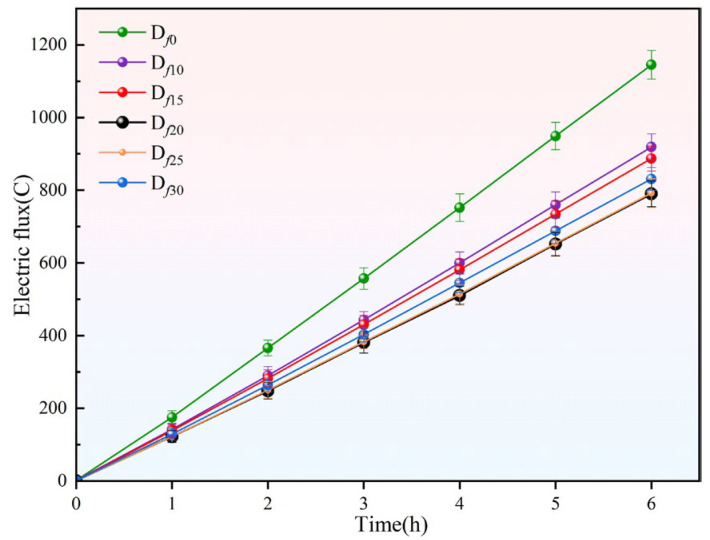
Electric flux test results.

**Figure 2:**
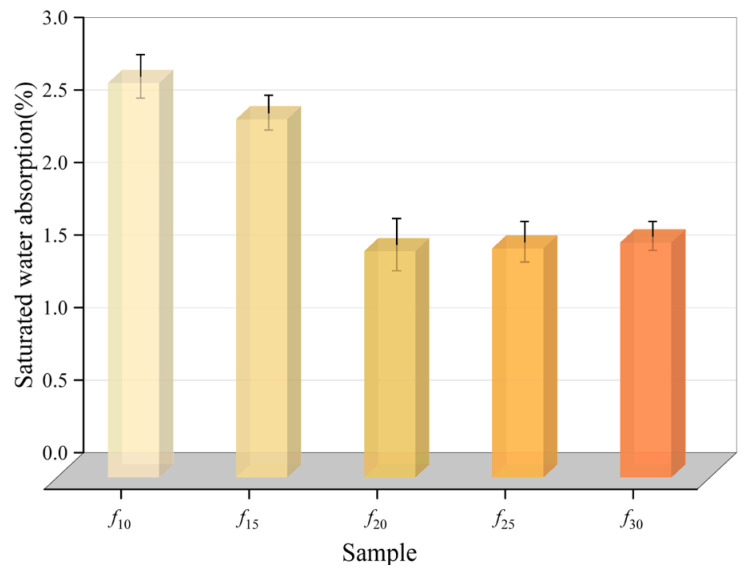
Saturated water absorption of the coating.

The water penetration resistance of the coating is assessed based on the electric flux test results and the saturated water absorption of the coating. The saturated water absorption ration (*W*_∞_) for each coating group is calculated using Equation (1), where *m*_0_ represents the mass of the coating before immersion and *m*_t_ is the mass of the coating when it is saturated with absorbed water. The results for these two parameters are shown in Figure 1 and Figure 2, respectively.

(1) $$W_{\infty }=\frac{m_{t}-m_{0}}{m_{0}}\times 100\%$$

During the electric flux testing, charge transport is facilitated by ions in the solution, with water acting as the medium for ion movement. By comparing and analyzing the electric flux test results for each group, the chloride ion penetration resistance of the coating on the concrete specimens can be assessed. As shown in Figure 1, after the application of the anticorrosion coating the electric flux on the concrete specimens decreased significantly. However, the improvement in the impermeability of the specimens varied. Among the coating groups, D*_f_*_20_ and D*_f_*_25_ showed the greatest improvement, with the electric flux decreasing by 31.0% and 30.8%, respectively, compared to the uncoated specimen D*_f_*_0_, followed by D*_f_*_30_ and D*_f_*_15_. D*_f_*_10_ showed the least improvement, with a 19.7% reduction in electric flux compared to D*_f_*_0_. Additionally, previous studies [19,20] have suggested that the diffusion properties of water directly influence the diffusion capacity of ionic solutions within the coating. Therefore, the water penetration rate can be inferred by analyzing the rate of increase in electric flux for each coating group. The electric flux for D*_f_*_0_ was the highest, followed by D*_f_*_10_, D*_f_*_15_, D*_f_*_30_, D*_f_*_25_, and D*_f_*_20_. As shown in the results, among the five coating groups tested, D*_f_*_20_ and D*_f_*_25_ exhibited the best resistance to permeability. These coatings hinder water transfer and diffusion, thereby slowing the chloride ion passage rate and resulting in a lower charge measurement.

As shown in Figure 2, the *W*_∞_ of *f*_10_ is the highest among all coating groups, being 1.10-, 1.74-, 1.72-, and 1.68-times that of *f*_15_, *f*_20_, *f*_25_, and *f*_30_, respectively. Notably, the saturated water absorption of *f*_20_ and *f*_25_ is lower than that of *f*_10_. This suggests that during the immersion test, *f*_10_ exhibited a higher water absorption, indicating a poor resistance to water penetration. In contrast, *f*_20_ and *f*_25_ demonstrated a superior water penetration resistance, effectively hindering water intrusion. These differences are closely related to the internal structure of the coatings, with factors such as micropores and hydrophilic molecular groups significantly influencing their water permeability resistance.

#### 3.1.2. Mechanical Properties of the Coating After Immersion

**Figure 3:**
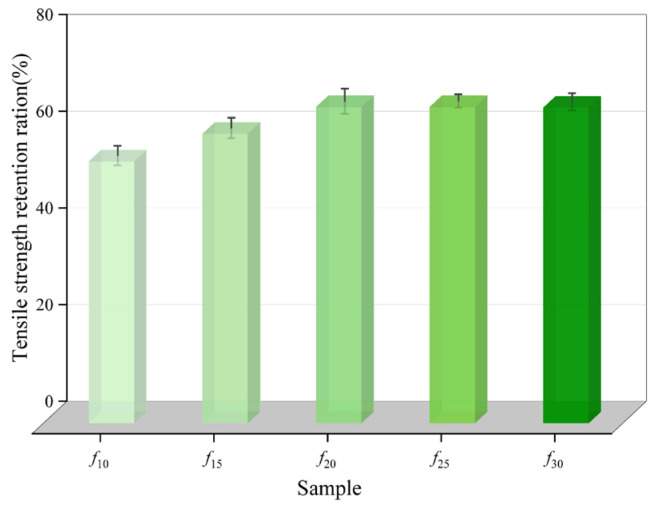
Tensile strength retention of the coating.

**Figure 4:**
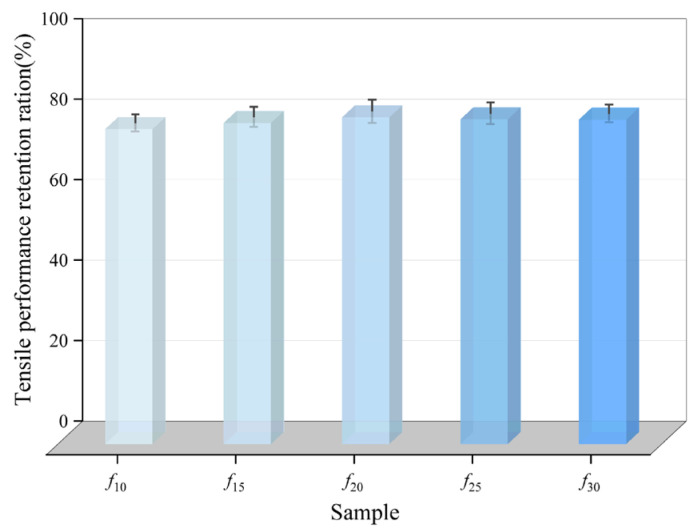
Retention ration of tensile properties of the coating.

Following the soaking treatment, the mechanical properties of the coatings deteriorated, as evidenced by a reduction in tensile strength and a decrease in plastic deformation capacity. The degree of deterioration in mechanical properties varied among the coatings due to differences in their water resistance. To quantitatively evaluate the deterioration of mechanical properties after water immersion, the tensile strength retention ration (*R*_T_) and tensile property retention ration (*R*_E_) were calculated based on the methods outlined in specification [21]. The specific calculation methods are as follows:

(2) $$R_{T}=\left(\frac{T_{1}}{T}\right)\times 100\%$$

(3) $$R_{E}=\left(\frac{E_{1}}{E}\right)\times 100\%$$

where *T*_1_ and *T* are the tensile strength of the coating after and before immersion, respectively. *E*_1_ and *E* denote the post-fracture elongation of the coating after and before water immersion, respectively. The calculated values of tensile strength retention ration (*R*_T_) and tensile property retention ration (*R*_E_) are shown in Figure 3 and Figure 4, respectively.

As shown in Figure 3 and Figure 4, as the diluent content increased the tensile strength retention ration (*R*_T_) of the coating initially increased, then plateaued, reaching a peak of 65.45% at *f*_25_. The tensile property retention ration (*R*_E_) for each coating group remained within the range of 78.30% to 81.19%, with minimal variation, and *f*_20_ exhibited the highest RE. Among both indicators, *f*_10_ exhibited the lowest values. Therefore, based on mechanical property retention, *f*_10_ demonstrates the weakest water resistance among the five coating groups, while *f*_20_ and *f*_25_ exhibit a superior water resistance.

In summary, water immersion leads to the degradation of the mechanical properties of the coating through two primary mechanisms after water infiltrates the coating [22,23]. The first mechanism is the plasticizing effect: when water molecules infiltrate the macromolecular chain segments, they preferentially form hydrogen bonds with hydrophilic groups in the polymer chains. This bonding reduces the original interactions between polymer chains, weakens cohesion, and leads to a reduction in mechanical properties, such as tensile strength. This effect is primarily influenced by the type and number of functional groups in the coating that can interact with water molecules. The second mechanism involves chemical reactions between water and specific molecular groups within the coating, resulting in the consumption of these groups and the subsequent fracture of molecular chains, further degrading the mechanical properties of the coating.

Based on the above analysis, it is clear that the water resistance of the coating is primarily governed by its resistance to water infiltration and the retention of its mechanical properties following infiltration. These factors are influenced by the microscopic pore structure governing water transport in the coating, as well as by characteristic functional groups, such as hydrophilic groups.

#### 3.1.3. Time-Varying State of Moisture in the Coating

Once water penetrates the coating, its distribution evolves over time. This time-dependent distribution can be analyzed to assess alterations in the internal structure of the coating and infer its degradation, indirectly reflecting the water resistance of the coating. This section analyzes the NMR test results of the coatings at different stages. The relaxation process involves energy exchange and release, with the relaxation time serving as an indicator of the speed of the process. Transverse relaxation, which is particularly sensitive to changes in the water state [24], is the primary focus, particularly the transverse relaxation time (*T*_2_) of each sample at various stages. *T*_2_ reflects both the chemical exchange between water and sample molecules, as well as the diffusion of water within distinct microenvironments. A smaller *T*_2_ value indicates a lower degree of freedom for water molecules and a stronger binding state [25].

**Figure 5:**
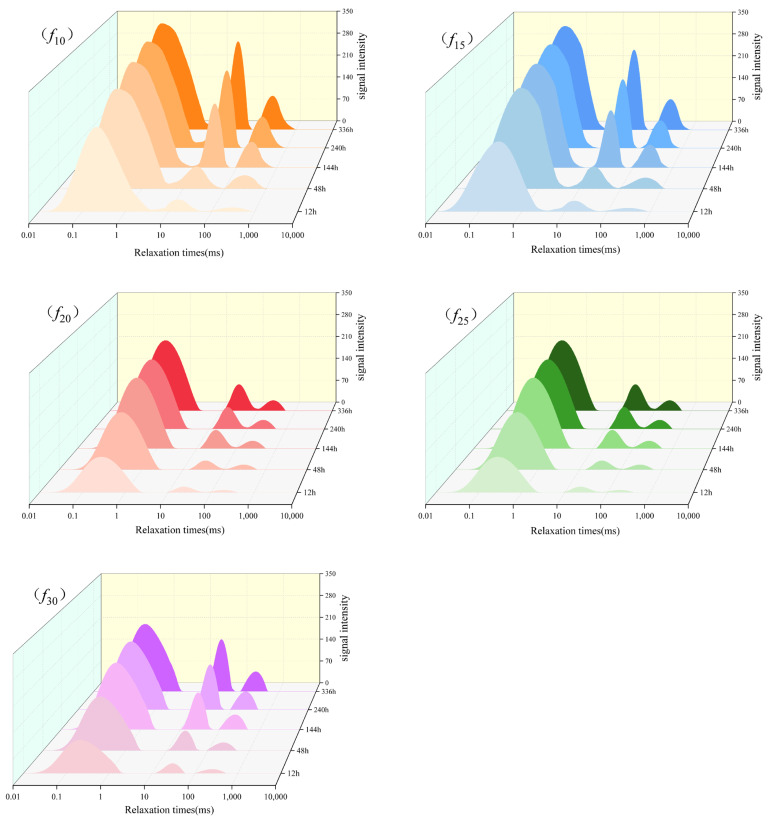
*T*_2_ test results of each coating group at different soaking ages.

The *T*_2_ test results for each coating sample at 0.5 days, 2 days, 6 days, 10 days, and 14 days are shown in Figure 5. As seen in Figure 5, each stage shows a primary peak and a secondary peak, located at approximately 0.30 ms and 35.00 ms, respectively. The *T*_2_ value of the primary peak band is primarily attributed to water molecules bonded to hydrophilic groups, with a *T*_2_ range of approximately 0 ms-to-10 ms, indicating the presence of bound water. The *T*_2_ value of the secondary peak band primarily reflects free water within the coating, with a range of approximately 10 ms-to-100 ms, indicating water in a free state. Throughout the test period, two forms of water—bound and free—persist in the coating. As soaking time increases, both the primary and secondary peaks shift to the right to varying extents, with the range of the secondary peak extending from 12.00 ms to 102.00 ms. This shift suggests an increase in the mobility of some free water within the coating, accompanied by a significant expansion of available storage space, likely due to microcracks formed as a result of coating deterioration.

**Figure 6:**
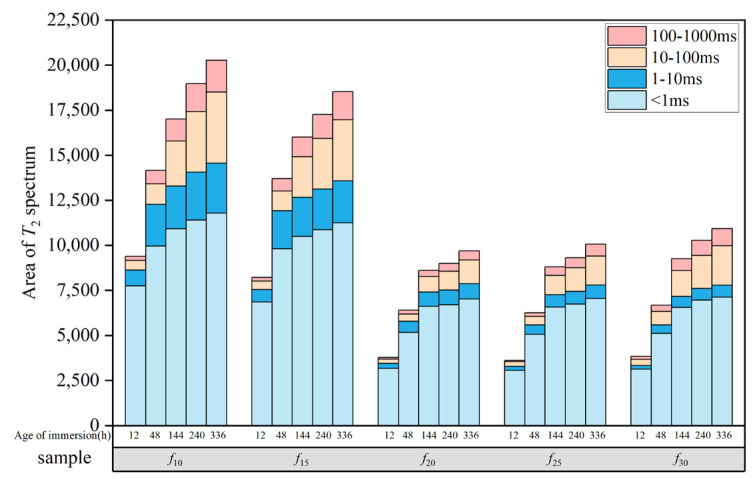
Moisture content of each coated sample at different *T*_2_ time intervals for different immersion ages.

The water content at different *T*_2_ time intervals was estimated semi-quantitatively based on the spectral peak areas, as shown in Figure 6. At each testing stage, the total amount of bound and free water was highest in sample *f*_10_, while samples *f*_20_ and *f*_25_ exhibited a relatively lower total water content. A comparative analysis of water content at 0.5 days and 20 days revealed a significant increase in both bound and free water content in *f*_10_, particularly the free water in the longer *T*_2_ time range, suggesting that more microcracks may have developed during the degradation process. In contrast, the increase in free water content in coatings *f*_20_ and *f*_25_ was smaller, suggesting fewer microcracks and a better resistance to water. This analysis also reveals that two distinct water infiltration processes occur during coating immersion: (1) water forms hydrogen bonds with hydrophilic groups, resulting in adsorption, and (2) water migrates through the micropores within the coating. These two modes of transmission are influenced by the distribution of hydrophilic groups and micropore structures in the coating.

### 3.2. Analysis of the Microscopic Properties of the Coating

The above analysis reveals significant differences in water resistance among coatings with varying diluent contents. This variation is primarily attributed to the microstructure and characteristic molecular groups of the coatings [26]. Variations in microscopic properties ultimately influence the macroscopic performance of the coating. To further investigate the mechanisms underlying water infiltration in specimens with different coatings, these two parameters should be examined for each coating group. Based on this analysis, the water resistance of the coatings can be assessed at the microscopic level.

#### 3.2.1. Microstructure

The microstructure influencing the water resistance of the coating primarily consists of micropores, which are distributed throughout the coating material as cavities, including both intra- and intermolecular pores. In this study, the “free volume” parameter is used to characterize the microstructure, specifically referring to the regions within the material that are unoccupied by molecules. This parameter represents a key defect in the material, reflecting its packing density and the strength of its chemical bonds [27,28]. This parameter largely governs the transport process of free water within the coating. The free volume in each coating was quantified using the positron annihilation lifetime (PALS) technique. Specifically, the annihilation of ortho-positronium (*o*-Ps) provides valuable insights into the microstructure of the sample, including pore size, distribution, and other defects [29]. Therefore, the analysis focuses on the *o*-Ps annihilation data.

**Figure 7:**
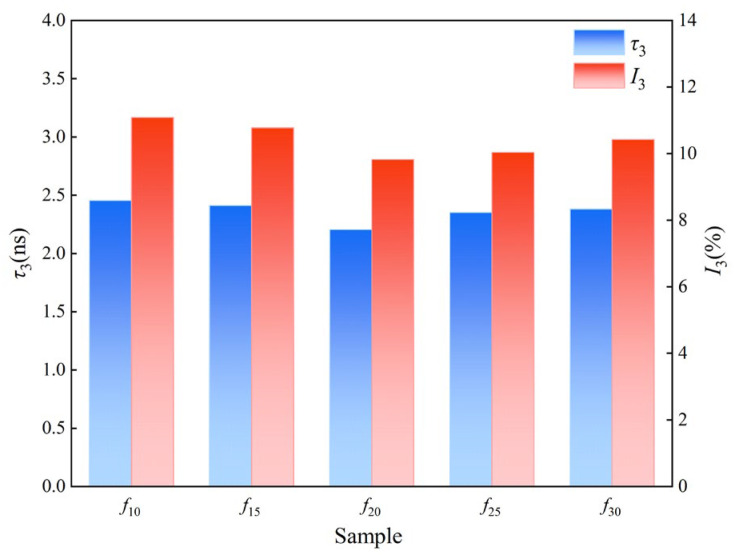
The *o*-Ps annihilation lifetime of each sample.

The positron annihilation lifetime spectra for each coating group were analyzed using a spectral solution program, yielding measurements of the annihilation lifetime (*τ*_3_) and intensity (*I*_3_) of *o*-Ps in the coatings, as shown in Figure 7. Figure 7 shows that as the diluent content in the coating increases, both *τ*_3_ and *I*_3_ values initially decrease and then increase, reflecting a corresponding trend in pore size and overall free volume content. Since *τ*_3_ and *I*_3_ reflect pore size and overall free volume content, respectively [30], the pore size and free volume content of the coating film initially decrease and then increase with a higher diluent content. This trend is mainly attributed to variations in the diluent amount, which affect the curing process and modify the microstructure of the resulting film. Furthermore, both excessive and insufficient diluent addition can hinder the curing reaction of the coatings. At low diluent levels, incomplete curing may occur due to premature curing of the surface film, which obstructs further curing within the material. Conversely, excessively high diluent levels introduce an abundance of small molecules, which can increase the size and content of the free volume within the cured film.

The mean pore volume of free volume (*V_f_*) and the free volume fraction (*F*_v_) were calculated to quantitatively assess the changes in the microstructure of the coating. In this context, *V_f_* and *F*_v_ represent the volume of a single free volume void and the total free volume content within the coating, respectively, and are determined using Equation (4) through (6).

(4) $$\tau_{3}=\frac{1}{2}{\left[1-\left(\frac{R}{R_{0}}\right)+\left(\frac{1}{2\pi }\right)\sin\left(\frac{2\pi R}{R_{0}}\right)\right]}^{-1}$$

(5) $$V_{f}=\frac{4}{3}\pi R^{3}$$

(6) $$F_{v}=CV_{f}I_{3}$$

**Figure 8:**
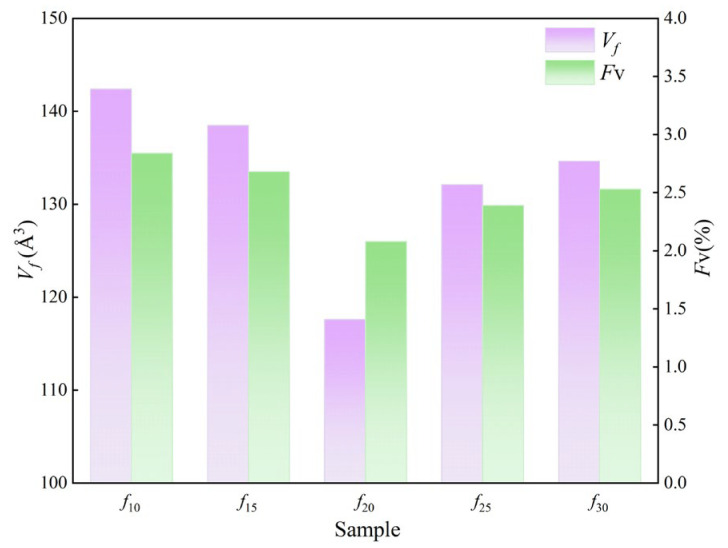
The average free volume radius and content of each sample.

In the equation above [31], *R* represents the free volume average aperture, expressed in Å units, as specified in Table 2. *R*_0_ = *R* + *ΔR*, where *ΔR* is the electron layer thickness, with a value of 1.66 Å; *C* is the structural constant, with a value of 0.0018. The values of *τ*_3_ and *I*_3_ are shown in Figure 7. The detailed calculation results of the free volume mean pore volume (*V_f_*) and free volume fraction (*F*_v_) are shown in Figure 8.

As shown in the figure above, both *V_f_* and *F*_v_ of each coating group decrease initially and then increase from *f*_10_ to *f*_30_. Among these, the *V_f_* and *F*_v_ values of *f*_10_ are the highest, whereas those of *f*_20_ are the lowest. Specifically, the *V_f_* of *f*_10_ is 1.03-to-1.21-times greater than that of the other groups, and the *F*_v_ is 1.06-to-1.37-times greater than that of the other groups. An increase in *V_f_* and *F*_v_ creates additional pathways for water molecule migration within the coating, thereby accelerating the diffusion rate of water molecules. Furthermore, when bonding sites within the coating become saturated with water molecules, any excess water remains as free water within the coating. These conditions may result in coating deterioration, significantly impairing its water resistance.

#### 3.2.2. Characteristic Molecular Groups

**Figure 9:**
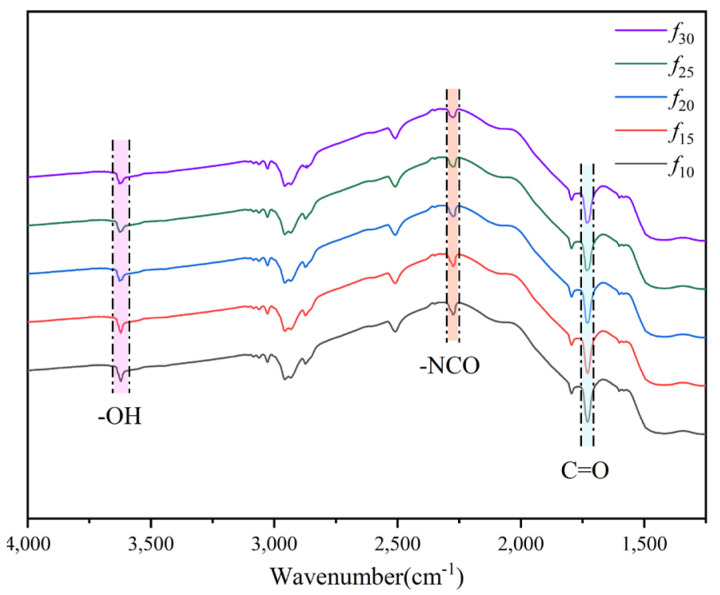
ATR−FTIR test results for each group of specimens.

**Figure 10:**
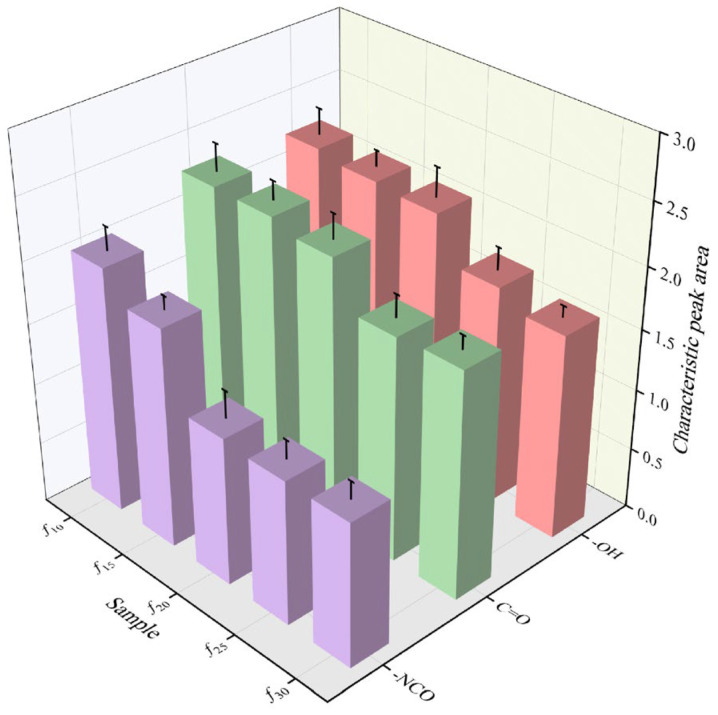
Characteristic peak areas of characteristic molecular groups for each group of samples.

Characteristic molecular groups, such as those exhibiting hydrophilicity, provide hydrogen-bonding sites for water molecules in the coating, allowing part of the infiltrated water to be stored as bound water. Meanwhile, some groups, such as ester groups, can hydrolyze with water molecules, leading to a deterioration in coating performance. Therefore, the type and quantity of these groups have a significant impact on the water resistance of the coating. In the polyurethane coatings examined in this study, the water-adsorbing molecular groups include hydroxyl and carbonyl groups [32,33]. Additionally, water chemically reacts with isocyanate groups in the coating, further compromising its mechanical properties [34]. To analyze these characteristic functional groups in different coatings, attenuated total reflection infrared spectroscopy (ATR−FTIR) was employed, with the test results shown in Figure 9. According to the Lambert−Beer law [35], the area of a characteristic peak can be used to semi-quantitatively determine the content of a molecular group. Therefore, this study uses the characteristic peak area of each functional group as an indicator of its content, with the specific calculation results shown in Figure 10.

As shown in Figure 10, *f*_10_ exhibits the highest content of all the aforementioned groups across all coating groups, whereas *f*_30_ shows the lowest content of hydroxyl and isocyanate groups, and *f*_25_ has the lowest carbonyl group content. The hydroxyl, carbonyl, and isocyanate group contents in *f*_10_ are 1.02-to-1.32-times, 1.00-to-1.24-times, and 1.12-to-1.71-times greater than those in the other groups, respectively. This is primarily due to the fact that the curing process of the polyurethane coating in this study requires moisture from the surrounding air. As the reaction progresses, the isocyanate groups in the coating decrease, while free hydroxyl and carbonyl groups form hydrogen bonds between molecular chain segments. As the diluent content gradually increases from *f*_10_ to *f*_30_, the curing rate of the coating slows, leading to a longer curing time for the surface layer. This extended curing time allows the inner coating to absorb more moisture from the air, promoting a more complete curing reaction and further reducing the residual isocyanate groups. Additionally, in the liquid state the molecular segments in the coating are more mobile, providing hydroxyl and carbonyl groups with additional time to form intermolecular hydrogen bonds. Consequently, the content of free hydroxyl and carbonyl groups decreases, reducing the available bonding sites after water infiltration, thereby further decreasing the residual content of these groups within the coating. These factors collectively enhance the water resistance of the coating to varying extents.

#### 3.2.3. Microanalysis of Water Resistance of Coatings

Based on the results presented in Section 3.1, the water resistance of the five coating groups investigated in this study varies and can be assessed through three key aspects: resistance to water penetration, retention of mechanical properties after immersion, and the time-dependent behavior of water within the coating. Resistance to water infiltration is crucial for ensuring the effective water resistance of coatings. Under real-world service conditions, minimal water penetration into the coating allows the microstructure and mechanical properties of the coating to remain largely unaffected. This allows coatings to retain their excellent mechanical properties and thermal stability, even in prolonged exposure to humid environments. However, organic coatings, such as polyurethane, cannot entirely prevent water infiltration, a phenomenon largely determined by their microstructure [36]. Water penetrates coatings primarily in two ways: first, it forms hydrogen bonds with hydrophilic groups in the coating, becoming bound water, which is then transferred through chain displacement [37]; second, it diffuses as free water through micropores and microcracks within the coating [38]. The first mode is primarily governed by the hydrophilic group content in the coating, whereas the second is influenced by the micropore content. Based on the results presented in Section 3.2.2, among the five coating samples investigated, *f*_10_ exhibits the highest content of two major hydrophilic molecular groups, along with the highest free volume content. As a result, *f*_10_ offers more bonding sites for water molecules and more channels for water transport, facilitating water penetration into the coating. This results in the highest levels of both bound and free water at various stages (as shown in Figure 5). In contrast, the two groups of coatings, *f*_20_ and *f*_25_, have a lower free volume content and hydrophilic groups, so it will be more difficult for water to hydrogen-bond with the coating when it comes into contact with the coating, and it will be difficult for free water molecules to be transported and diffused within the coating. As a result, these two coatings display a superior resistance to water infiltration. This difference accounts for the varying impermeability observed in each coating group in Section 3.1.

**Figure 11:**
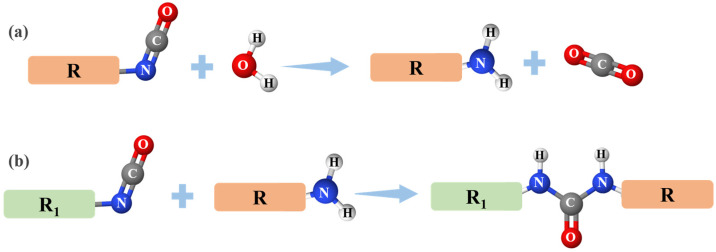
Curing reaction process of polyurethane coating: (**a**) -NCO reacts with H_2_O to form a binary or polyamine, (**b**) the amine reacts with unreacted –NCO in a chain expansion reaction.

When water penetrates the coating, it affects the material in two primary ways. First, through its plasticizing effect [22] water reduces the cohesion between macromolecular chain segments, leading to a decrease in the strength of the coating. Second, water reacts with ester groups in the coating, initiating hydrolysis that breaks the main chains, increases the free volume between molecular chains, alters the spatial arrangement of chain segments, and reduces their regularity, ultimately diminishing tensile strength and post-fracture elongation of the coating [23]. Furthermore, the coating studied in this work is a single-component, moisture-cured polyurethane, with its curing reaction process shown in Figure 11. Once water infiltrates the coating, it can also react with free isocyanate groups, releasing carbon dioxide. This reaction results in an increase in internal voids, depletion of isocyanate groups, and creation of additional space for water storage, thereby accelerating the degradation of the mechanical properties of the coating [39]. According to the functional group test results in Section 3.2.2, *f*_10_ has the highest initial content of free isocyanate groups, leading to the greatest decrease in isocyanate content after water immersion, which explains why its tensile strength retention ration (*R*_T_) is the lowest.

**Figure 12:**
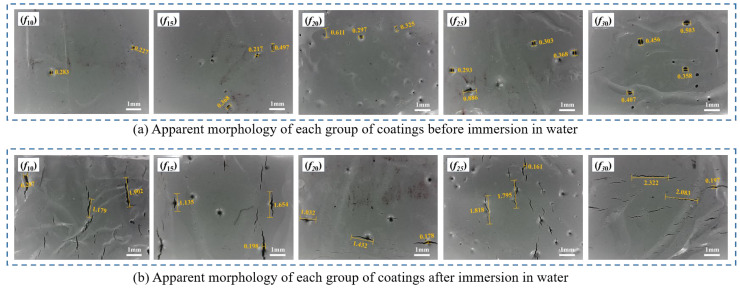
The apparent morphology of each group of coatings before and after water resistance deterioration.

In addition to the aforementioned effects, water penetration induces defects, primarily cracks, in the coating due to a combination of physical and chemical factors, such as swelling from water absorption and ester group hydrolysis [40]. The crack observation instrument (ZBL-F130) was used to characterize the mesostructural features and surface morphology of each coating group, as shown in Figure 12. It can be seen from Figure 12 that before degradation both the number and size of pores on the coating surface increase with increasing diluent content. These pores form as a result of the rapid volatilization of the diluent, which creates bubbles that cannot dissipate promptly during curing [41]. After degradation, the number, length, and width of surface cracks initially increase and then decrease with increasing diluent content, with *f*_20_ and *f*_25_ exhibiting lower degrees of degradation, consistent with the macroscopic water performance indices. This behavior is influenced by two primary factors: (i) Prior to coating degradation, surface pores increase with increasing diluent content, affecting coating integrity, facilitating water infiltration, and consequently reducing water resistance. (ii) As indicated by the results in Section 3.2.2, the content of characteristic molecular groups (hydroxyl, carbonyl, and isocyanate) gradually decreases with increasing diluent content, leading to a reduced resistance to water infiltration and, consequently, improving water resistance. These combined effects result in a trend where surface cracks first increase and then decrease with increasing diluent content. These cracks and pores concentrate stress during tensile testing, thereby reducing tensile strength and increasing the likelihood of brittle fracture, which limits plastic deformation and contributes to a decrease in tensile property retention (*R*_E_). Furthermore, the cracks and pores provide additional pathways for water infiltration, accelerating hydrolysis and further diminishing the water resistance of the coating.

The above analysis demonstrates that when the coating exhibits a strong resistance to water infiltration, the plasticizing effect is reduced as a result of decreased water penetration under identical immersion conditions. Consequently, the amount of water available for hydrolysis with ester groups diminishes, slowing the hydrolysis process and ultimately improving the water resistance of the coating. Furthermore, it can also be observed that the water resistance of the coating is primarily influenced by its microporous structure and the content of characteristic molecular groups, such as hydrophilic groups. Based on the above analysis, the water resistance of the *f*_20_ and *f*_25_ coatings is superior to that of the other coatings.

## 4. Preferential Analysis of Water Resistance of Coatings

The analysis above illustrates that the microstructural state of the coating significantly influences its macroscopic performance. The water resistance of the coating depends on multiple micro-level indicators, while the overall water resistance is represented by various macro-level indicators [42]. To further examine the correlation between the diluent content of the coating and its resistance to water, this study applied the entropy weight method and equal weight method to comprehensively evaluate water resistance, incorporating both macroscopic and microscopic performance indicators. Among them, the entropy weight method utilizes index data effectively, minimizing the influence of subjective factors and assigning objective weights to the variables under investigation [43,44]. Based on the calculation procedures described in relevant studies [44], seven coating indicators—electric flux, saturated water absorption ration, tensile strength retention ration, tensile property retention ration, free volume mean pore volume, free volume fraction, and characteristic molecular groups content—were weighted and scored. Of these, electric flux, saturated water absorption ration, tensile strength retention ration, and tensile property retention ration are macroscopic test indicators, while free volume mean pore volume, free volume fraction, and characteristic molecular groups content represent microscopic performance indicators. The specific data used in the calculation process are provided below:

Step 1: The normalization of various indicators. Since there are significant differences in the content and dimensions of each indicator of the coating, it is necessary to first perform dimensionless treatment. Its standardization is calculated using Equation (7), and the calculation results are given in Table 3.

(7) $$P_{ij}=\frac{x_{ij}-minx_{ij}}{maxx_{ij}-minx_{ij}}$$

Step 2: The weight of the sample for each indicator was calculated using Equation (8), and the results are given in Table 4.

(8) $$R_{ij}=\frac{P_{ij}}{\sum_{i=1}^{n}P_{ij}}$$

Step 3: Based on the entropy weight method, the entropy value, redundancy, and weight of each indicator were determined, calculated using Equation (9) through (12), respectively, with the results given in Table 5. Meanwhile, the weight of each indicator was determined based on the equal weight method, as given in Table 6.

(9) $$E_{j}=-k\sum_{i=1}^{n}P_{ij}ln(P_{ij})$$

(10) $$k=\frac{1}{\ln\left(n\right)}$$

(11) $$d_{j}=1-E_{j}$$

(12) $$w_{j}=\frac{1-E_{j}}{\sum_{j=1}^{m}\left(1-E_{j}\right)}$$

**Figure 13:**
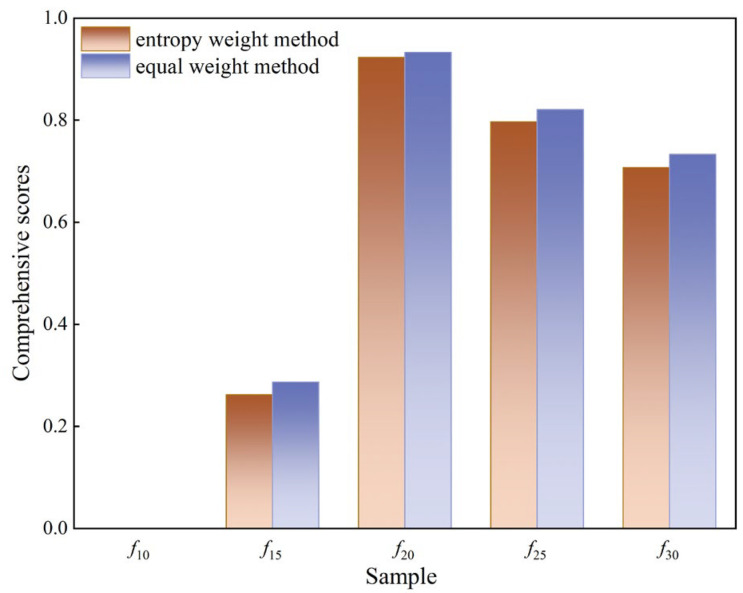
Comprehensive scores for each sample of the entropy weight method and equal weight method.

Step 4: The comprehensive score of each sample was calculated according to the data in Table 5 and Table 6 using Equation (13), and the calculation results are shown in Figure 13.

(13) $$S_{i}=\sum_{j=1}^{m}w_{j}x_{ij}$$

As shown in Figure 13, the results of the entropy weight method and equal weight method exhibit good consistency, with only a small difference. Both methods indicate that the combined score of each specimen, calculated using macro test indices and micro performance indices, is as follows, in descending order: *f*_20_ > *f*_25_ > *f*_30_ > *f*_15_ > *f*_10_. Furthermore, it can also be observed that, when considering both macro test indices and micro performance indices, with an increase in diluent content, the water degradation resistance of the coating first increases and then decreases. Notably, *f*_20_ and *f*_25_ exhibit a better water degradation resistance, making them more suitable for protective applications requiring a high water resistance. The water degradation resistance of *f*_25_ is superior, making it more suitable for protection projects with high water resistance requirements.

## 5. Conclusions

(1) From the perspective of water penetration resistance, mechanical property retention after water immersion, and time-dependent water distribution, it is observed that, among the five coatings studied, *f*_10_ exhibits the poorest water resistance, while *f*_20_ and *f*_25_ demonstrate a superior resistance to water. The order is as follows: *f*_20_ > *f*_25_ > *f*_30_ > *f*_15_ > *f*_10_.
(2) With increasing water immersion time, the content of bound and free water shows distinct variations among the coatings. These variations are primarily attributed to changes in the mesostructure and microstructure of the coatings, as well as the content of characteristic molecular groups, including hydrophilic groups. Notably, the increase in free water content is more pronounced, primarily resulting from crack formation due to coating deterioration.
(3) With an increase in diluent content, the average free volume pore volume and free volume fraction of polyurethane coatings initially decrease and then increase. Simultaneously, the content of characteristic molecular groups, such as hydrophilic groups, gradually decreases. Under the combined influence of these two factors, the water resistance of the coatings initially improves and then deteriorates as diluent content increases.
(4) Using the entropy weight method and equal weight method, combined with macro water resistance test indices and micro performance indices, the comprehensive scores for each sample were calculated as follows (in descending order): *f*_20_ > *f*_25_ > *f*_30_ > *f*_15_ > *f*_10_. The water resistance of the coatings exhibited a trend of first increasing and then decreasing as diluent content increased. Specifically, *f*_20_ and *f*_25_ demonstrated the best water resistance performance.

## Figures and Tables

**Table 1:** Mix proportions of concrete.

Water	Cement	Sand	Crushed Stone	Water-Reducing Admixture
/(kg/m^3^)	/(kg/m^3^)	/(kg/m^3^)	/(kg/m^3^)	/%
150	395	766	1149	1.3

**Table 2:** The average free volume pore diameter of each coating group.

Sample Number	*f*_10_	*f*_15_	*f*_20_	*f*_25_	*f*_30_
*R*/Å	3.24	3.21	3.04	3.16	3.18

**Table 3:** The values of every index for the coating post-normalization treatment.

Sample Number	Electric Flux	Saturated Water Absorption	Tensile Strength Retention Ration	Tensile Retention Ration	Free Volume Mean Pore Volume	Free Volume Fraction	Characteristic Molecular Group Content
*f*_10_	0	0	0	0	0	0	0
*f*_15_	0.248	0.216	0.504	0.517	0.158	0.211	0.152
*f*_20_	1.000	1.000	0.991	1.000	1.000	1.000	0.535
*f*_25_	0.984	0.983	1.000	0.828	0.415	0.592	0.941
*f*_30_	0.682	0.948	0.982	0.793	0.313	0.408	1.000

**Table 4:** The proportion of the sample under each index.

Sample Number	Electric Flux	Saturated Water Absorption	Tensile Strength Retention Ration	Tensile Retention Ration	Free Volume Mean Pore Volume	Free Volume Fraction	Characteristic Molecular Group Content
*f*_10_	0	0	0	0	0	0	0
*f*_15_	0.085	0.068	0.145	0.165	0.084	0.095	0.058
*f*_20_	0.343	0.318	0.285	0.319	0.530	0.452	0.203
*f*_25_	0.338	0.312	0.288	0.264	0.220	0.268	0.358
*f*_30_	0.234	0.301	0.282	0.253	0.166	0.185	0.381

**Table 5:** Entropy, redundancy, and weight of each index of the entropy weight method.

Sample Number	Electric Flux	Saturated Water Absorption	Tensile Strength Retention Ration	Tensile Retention Ration	Free Volume Mean Pore Volume	Free Volume Fraction	Characteristic Molecular Group Content
Entropy value	0.797	0.791	0.841	0.845	0.730	0.775	0.760
Redundancy	0.203	0.209	0.159	0.155	0.270	0.225	0.240
Weight	0.139	0.143	0.109	0.106	0.185	0.154	0.164

**Table 6:** Weight of each index of the entropy weight method.

Sample Number	Electric Flux	Saturated Water Absorption	Tensile Strength Retention Ration	Tensile Retention Ration	Free Volume Mean Pore Volume	Free Volume Fraction	Characteristic Molecular Group Content
Weight	0.143	0.143	0.143	0.143	0.143	0.143	0.143

## Data Availability

Data are contained within the article.
